# Enhanced Specificity of TPMT*2 Genotyping Using Unidirectional Wild-Type and Mutant Allele-Specific Scorpion Primers in a Single Tube

**DOI:** 10.1371/journal.pone.0091824

**Published:** 2014-04-04

**Authors:** Dong Chen, Zhao Yang, Han Xia, Jun-Fu Huang, Yang Zhang, Tian-Nun Jiang, Gui-Yu Wang, Zheng-Ran Chuai, Wei-Ling Fu, Qing Huang

**Affiliations:** 1 Department of Laboratory Medicine, Southwest Hospital, Third Military Medical University, Chongqing, P. R. China; 2 Department of Blood Transfusion, Southwest Hospital, Third Military Medical University, Chongqing, P. R. China; Institut Pasteur of Shanghai, Chinese Academy of Sciences, China

## Abstract

Genotyping of thiopurine S-methyltransferase (TPMT) is recommended for predicting the adverse drug response of thiopurines. In the current study, a novel version of allele-specific PCR (AS-PCR), termed competitive real-time fluorescent AS-PCR (CRAS-PCR) was developed to analyze the TPMT*2 genotype in ethnic Chinese. This technique simultaneously uses wild-type and mutant allele-specific scorpion primers in a single reaction. To determine the optimal conditions for both traditional AS-PCR and CRAS-PCR, we used the Taguchi method, an engineering optimization process that balances the concentrations of all components using an orthogonal array rather than a factorial array. Instead of running up to 264 experiments with the conventional factorial method, the Taguchi method achieved the same optimization using only 16 experiments. The optimized CRAS-PCR system completely avoided non-specific amplification occurring in traditional AS-PCR and could be performed at much more relaxed reaction conditions at 1% sensitivity, similar to traditional AS-PCR. TPMT*2 genotyping of 240 clinical samples was consistent with published data. In conclusion, CRAS-PCR is a novel and robust genotyping method, and the Taguchi method is an effective tool for the optimization of molecular analysis techniques.

## Introduction

Since their invention [1], thiopurine drugs, including azathioprine (AZA), 6-mercaptopurine (6MP), and 6-thioguanine (6TG), have significantly advanced the treatment of hematologic malignancies, organ transplantation, inflammatory bowel disease (IBD) and some autoimmune diseases (e.g., rheumatoid arthritis, autoimmune hepatitis) [2]. Thiopurine drugs are metabolized by thiopurine methyltransferase (TPMT) [2], which may have single nucleotide polymorphisms (SNPs), resulting in null or decreased enzyme activity [1], [2], [3], [4], [5]. Since the initial identification of SNPs in TPMT, more than 30 mutant alleles of the gene have been identified, of which TPMT*2, *3A, *3B, *3C, and *4 are the most common [1], [2], [3], [4], [5]. About 3–14% of patients have a heterozygous TPMT genotype with one variant TMPT allele [1], [2], [3], [4], [5]. Independent and professional groups have recommended analyzing the TPMT genotype status to determine thiopurine drug dosing [5], [6], [7], [8], [9]. Accordingly, the Clinical Pharmacogenetics Implementation Consortium (CPIC) recommends that the initial dosage of AZA or 6-MP be reduced by 30–70% for patients who possess heterozygous genotypes of TPMT*2, *3A, *3C, or *4 [6], [7]. Therefore, TPMT genotyping may be applicable for personalized therapy to predict the adverse response to thiopurine drugs.

Among the methods of TPMT genotyping, restriction-fragment length polymorphism (RFLP)-southern blot assay, RFLP-PCR, oligonucleotide ligation assay, and allele-specific PCR (AS-PCR) or the amplification-refractory mutation system (ARMS) have been used. AS-PCR is one of the most common methods and uses (AS) primers or probes labeled with fluorescent reporter dyes (usually hydrolysis probes or dual hybridization probes) to identify the genotypes of the targeted SNPs [10], [11], [12], [13], [14], [15]. Because only a single nucleotide difference exists between the defined and opposite genotypes, allelic determination is often hampered by cross-hybridization between either defined genotypic probes and opposite amplicons, or defined genotypic AS primers and opposite templates [16], [17], [18], [19], [20]. Consequently, stringent reaction conditions (e.g., high annealing temperatures, low AS primer concentrations) are needed to avoid non-specific genotypic determination. However, the optimization of traditional AS-PCR is costly and time-consuming, and optimization cannot always completely eliminate non-specific amplification (7). Therefore, novel strategies to avoid the non-specific genotypic determination of AS-PCR are needed.

Generally, molecular biologists optimize assays using factorial methods, which involve testing all the levels of all factors against one another, requiring numerous experiments [21], [22], [23]. As an alternative approach to reduce time and effort, the Taguchi method [21], [22], [23], was first applied to molecular biological methodologies for the optimization of traditional end point PCR in 1994 [24]. The Taguchi method has also been used for DNA amplification fingerprinting [25], end-point multiplex PCR [26], [27], [28], real-time quantitative PCR using SYBR Green chemistry [29] and fluorescence resonance energy transfer (FRET) probes [30]. Although this method has been effective for several molecular assays, it has yet to be demonstrated for AS-PCR [31].

The Taguchi method has been widely used in automotive and electronics industrial design, principally in development trials, for the establishment of optimal conditions for a particular process using a minimal number of experiments [28], [32], [33], [34], [35]. This method is applied towards experimental objectives that are “nominal-the-best”, “smaller-the-better”, “larger-the-better”, or “zero-point proportional” [21], [22], [23]. Quality loss functions (signal-to-noise ratios; *S/N* ratios) penalize deviations from prediction values, and consequently, orthogonal arrays can be designed to examine multiple factors in few experimental trials [21], [22]. Thus, a trial investigating the effects and interaction of five PCR reaction components (control factors) each at four levels would require an experiment with 1024 (i.e., 4^5^) separate experiments if tested by traditional array design. Using the Taguchi method, an estimate of the effect of each component could be arranged into an L_16_(4^5^) orthogonal array that only requires 16 experiments.

In the present study, a novel method of AS-PCR, termed competitive real-time fluorescent AS-PCR (CRAS-PCR), was developed to analyze the genotypes of the TPMT*2 variant (dbSNP: rs1800462G>C; p.A80P; c.238G>C; g.11421G>C). This method uses wild-type allele-specific forward (WT-ASF) and mutant allele-specific forward (MT-ASF) scorpion primers in a single reaction mixture. The Taguchi method was used to optimize reaction components and evaluate reaction characteristics of both traditional AS-PCR and CRAS-PCR. Compared with traditional AS-PCR, non-specific amplification can completely be eliminated in CRAS-PCR following optimization by the Taguchi method. Preliminary application of CRAS-PCR in the analysis of 240 clinical samples further confirmed CRAS-PCR as a routine, reliable and simple method to analyze SNP genotypes, including the TPMT*2 variant.

## Materials and Methods

### Materials and equipment

The traditional end-point PCR and real-time PCR instruments used in this study were the GeneAmp PCR System 2700 (Applied Biosystems, Foster City, CA, USA) and Exicycler™ 96 Real-time Quantitative Thermal Block (Bioneer, Daejeon, Korea). Real-time PCR data were collected and analyzed using Exicycler™ 96 Software (Bioneer). Ex Taq Hot Start Version DNA Polymerase (TaKaRa, Dianian, China) and a MutanBEST Kit (TaKaRa) were used to construct wild-type quality control (WT-QC) and mutant-type quality control (MT-QC) plasmids. Real-time PCR was performed with 2× master mix for Premix Ex Taq (Perfect Real Time; TaKaRa). QC plasmids were sequenced using a BigDye Terminator V3.1 Cycler Sequencing Kit (Applied Biosystems, Foster City, CA, USA) and the ABI Prism 3500 Genetic Analyzer (Applied Biosystems). Oligonucleotide sequences were synthesized at Sangon Biotech Co., Ltd. (Shanghai, China; Table 1). Genomic DNA of human whole blood was extracted using the QIAamp DNA Blood Mini Kit (QIAGEN GmbH, Hilden, Germany). GelRed (10,000×; Biotium Inc., Hayward, CA, USA) was diluted to 100× concentrations in 6× Loading Buffer (TaKaRa, Dalian, China) for nucleic acid staining following agarose gel electrophoresis [36]. Images of stained gels were captured with a UV transilluminator (Vilber Lourmat, Marne la Valled, France).

**Table 1:** Oligonucleotides used in the present study.

ID	Descriptions	Sequences (5′→3′)
HQ-589	Exon-4 F	ATAACCCTCTATTTAGTCATTTG
HQ-590	Exon-4 R	TGGTATCCTCATAATACTCACA
HQ-654	WT-ASF	(CY5)**ACCGCGC**CACCAACTACACTGTGTCCC**GCGCGGT**(BHQ2)(HEG)AATGTATGATTTTATGCAGGTTA *G*
HQ-645	MT-ASF	(FAM)**ACCGCGC**CACCAACTACACTGTGTCCC**GCGCGGT**(BHQ1)(HEG)GTATGATTTTATGCAGGTTC *C*
HQ-640	CO-R	TACCCAAATCAAAACAAACC

The positions refer to the primer regions located in the TPMT reference genomic DNA (GenBank ID: NG_012137.1). HQ-654 and HQ-645 are scorpion primers specifically targeting the WT and MT TPMT*2 alleles, respectively. The stem-loop structure is marked with underlined bold letters. The position of labeled fluorescent reporter (6-FAM or CY5), the corresponding quencher (BHQ1 or BHQ2), and PCR amplification blocker (HEG) are indicated. The underlined letters in the *penultimate* (second to the terminal) position indicate the mismatch introduced to increase AS primer specificity according to the WASP principle. The 3′-end terminal italicized letters indicate allele-distinguishable variants.

### Samples and genomic DNA extraction

This study was approved by the institutional review board and ethics committee of Southwest Hospital (Chongqing, China). Human blood samples were collected from ethnic Han Chinese volunteers living in the Chongqing territory. Written informed consent was obtained prior to collection. Coagulation of the blood samples was prevented with dipotassium ethylenediamine tetraacetic acid (K2-EDTA, 1.5 mg/ml blood). Genomic DNA was extracted using the QIAamp DNA Blood Mini Kit (Qiagen GmbH) according to the manufacturer's instructions. The genomic DNA concentration of each clinical sample was determined using the Quant-iT™ PicoGreen dsDNA Assay Kit (Invitrogen, Carlsbad, CA, USA) according to the manufacturer's instructions.

### Construction of quality control (QC) plasmid

The PCR reaction mixture (20 μl) used to amplify whole exon 4 fragments containing TPMT*2 included 1× Ex Taq Buffer (Mg^2+^ plus; TaKaRa), 250 μM of each dNTP (TaKaRa), 0.5 units of Ex Taq HS (TaKaRa), 200 nM each of forward and reverse primer (HQ-589 and HQ-590 in Table 1) and 20 ng of human genomic DNA. Reactions were performed as follows: denaturation at 94 °C for 5 min; 35 cycles of 94 °C for 30 sec, 55 to 65 °C for 30 sec and 72 °C for 30 sec; and final extension at 72 °C for 5 min. DNA sequencing of the PCR products was performed using the BigDye Terminator V3.1 Cycler Sequencing Kit (Applied Biosystems). All sequencing analyses were performed at least twice in duplicate, and both forward and reverse sequencing were performed. After the wild-type allele of TPMT*2 was confirmed, the PCR products were cloned to construct the WT-QC plasmid. The WT-QC plasmid was subsequently used to prepare the MT-QC plasmid using the MutanBEST Kit (TaKaRa) according to the manufacturer's instructions.

### Real-time PCR

Three types of primers were designed: WT-ASF and MT-ASF scorpion primers targeting WT and MT TPMT*2 alleles, and a common reverse (CO-R) primer (Table 1). For each Assay Type, 1×10^4^ copies were used of plasmids WT-QC, MT-QC, or mixed QC (MIX-QC; an equal ratio of WT- and MT-QC).

The real-time PCR mixture (20 μl) contained 1× Premix Ex Taq master mix (Perfect Real Time; TaKaRa), one of the three QC plasmid types, and variable concentrations of primers (Table S1 to S3). Reactions were performed with the following cycling conditions: initial denaturation of 95 °C for 30 sec; followed by 40 cycles of 95 °C for 30 sec and the desired annealing temperature (Table S1) for 30 sec (with single fluorescence acquisition). All temperature transition ramping rates were at 2.5°C/sec. The quantification cycle (*C*_q_) values were automatically determined by Exicycler™ 96 Software (Bioneer). All reactions were performed at least in duplicate with no-template control to monitor for contamination and non-specific products.

### The Taguchi method

The cycle number at which the fluorescence significantly differs from the background noise (the *C*_q_) is inversely proportional to the *log* of the initial quantity of DNA [16], [37], [38]. For each of the three Assay Types (Table 2), the *C*_q_ was selected as the response variable, except for Assay No. 3-3, for which the difference in *C*_q_ values (Δ*C*_q_) between WT and MT was selected as the response variable. Four factors were optimized at four levels (Table S1); therefore, according to the Taguchi method, an L_16_(4^5^) orthogonal array was designed (Table S2, S3). All calculations of the Taguchi method were done by inputting the formulae described below (Eq. 1–8) and corresponding values into a Microsoft Excel spreadsheet.

**Table 2:** Detailed information of components evaluated in each assay.

Assay Type	Assay No.	Control Factors				Signal Factors		
		WT-ASF	MT-ASF	CO-R	Tm	WT-QC plasmid	MT-QC plasmid	MIX-QC plasmid
1	1-1	√	×	√	√	√	×	×
	1-2	√	×	√	√	×	√	×
	1-3	√	×	√	√	×	×	√
2	2-1	×	√	√	√	√	×	×
	2-2	×	√	√	√	×	√	×
	2-3	×	√	√	√	×	×	√
3	3-1	√	√	√	√	√	×	×
	3-2	√	√	√	√	×	√	×
	3-3	√	√	√	√	×	×	√

An optimized assay should have the lowest possible *C*_q_ (or the lowest possible Δ*C*_q_ for Assay No. 3-3); therefore, the “smaller-the-better” equation (Eq. 1) was employed to calculate the S/N ratio (*η*) using an experimentally determined *C*_q_, except for Assays No. 1-2 and 2-1. For these assays, the “larger-the-better” equation (Eq. 2) was used to evaluate and avoid the non-specific amplification of WT-ASF and MT-ASF primers on opposite templates. In the absence of an amplification signal (i.e., no *C*_q_ values determined by Exicycler™ 96 Software), a manual *C*_q_ value of 40 was assigned. The “smaller-the-better” and “larger-the-better” *S*/*N* ratio were calculated as follows:
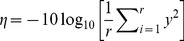
(1)

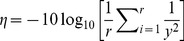
(2)where *η* =  the *S*/*N* ratio, *r* = the number of repeated runs, *y* = the response (*C*_q_ or Δ*C*_q_).

The percent contribution (*P*_C_) of each factor to the total variations observed in each experiment was calculated as follows [21] (Eq. 3 to 5):
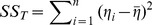
(3)

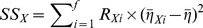
(4)

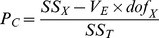
(5)where *SS*_T_ = the total sum of squares, *n* = the number of experiments in the orthogonal array, *η*_i_ = the *S*/*N* ratio of each experiment, and 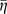 = the average *S*/*N* ratio of all experiments; *SS*_X_ = the sum of squares of the signal-to-noise ratio of factor X, in which *f* = the number of factors, *R*_Xi_ is the number of runs conducted at level *i* of factor *X*; 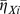 = the average *S*/*N* ratio for each level of each factor; *V*_E_ = the variance of error; *dof*_X_ = the degree of freedom of factor *X*. The *F*-ratio and *p*-value of each factor were further analyzed by Analysis of Variance (ANOVA).

To predict the *S*/*N* ratio of a reaction carried out with optimal conditions, the following equation was used [21] (Eq. 6):
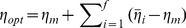
(6)where *η*_m_ = the overall mean of *S*/*N* ratios at the optimal level for all factor combined, 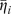 = the mean *S*/*N* ratio at optimal levels of each factor *i*.

The 95% confidence interval at optimal conditions was calculated as follows [21] (Eq. 7):
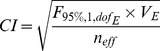
(7)where *F*_(95%,1,*dof*E)_ = the first *F* value with the degree of freedom equal to 1 and the degree of freedom of error (*dof*_E_) as the second degree of freedom at 95% confidence; *V*_E_ = the variance of error; *n*_eff_ =  the effective sample size determined by N/(1+*dof*_TF_), where N = the total number of experiments, and *dof*_TF_ = the degree of freedom of all factors combined.

As confirmation, the *S*/*N* ratio should fall within the 95% confidence interval, given that a prediction model is suitable. The confidence interval was calculated using the following equation [21] (Eq. 8):
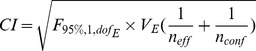
(8)where *n*_conf_ = the number of confirmatory tests conducted.

### Preliminary application of the CRAS-PCR system to clinical samples

WT-, MT- and MIX-QC plasmids at 10-fold serial dilutions (i.e., 1×10^8^ to 1×10^1^ copies) were further used to evaluate optimized CRAS-PCR conditions obtained from the Taguchi method. The optimized CRAS-PCR conditions were then used to analyze the TPMT*2 genotype of 240 clinical samples in which 50 to 150 ng of genomic DNA were used in reaction mixtures. Using a conversion factor of 6.6 pg per diploid human cell [39], [40], this concentration equals 1.5×10^4^ to 4.5×10^4^ copies of the corresponding genotypic allele. The *C*_q_ values of each sample were assessed within CY5 and 6-FAM fluorescent channels, indicating wild-type and mutant alleles, respectively. To confirm the results of CRAS-PCR, an exon 4 fragment containing TPMT*2 for each sample was amplified and both forward and reverse sequencing were carried out (Table 1) as described in previous sections.

## Results

### The principles of CRAS-PCR

**Figure 1:**
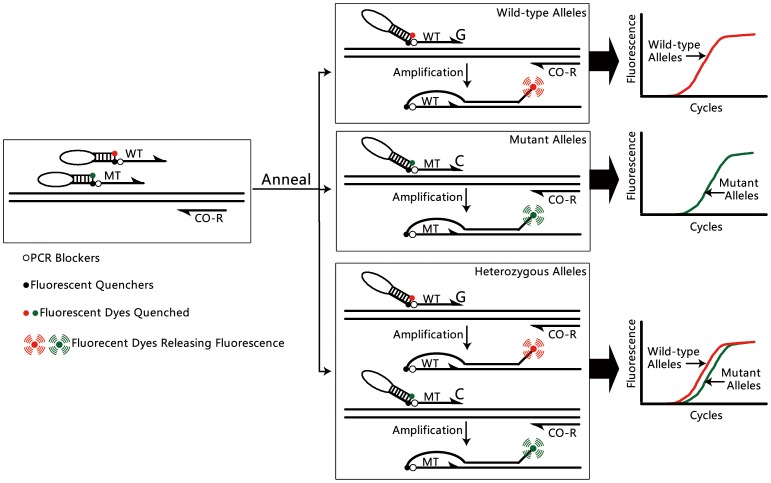
Principle of CRAS-PCR using unidirectional competitive AS scorpion primers. The WT and MT oligonucleotides refer to WT-ASF and MT-ASF primers, respectively. The WT-ASF, MT-ASF and CO-R primers depicted in the left panel were included in a single reaction. The middle panel shows the predicted reaction response for WT homozygotes, MT homozygotes, and WT/MT heterozygotes. Their corresponding amplification curves are indicated in the right panel, for which the red (CY5 fluorescent channel) and green (6-FAM fluorescent channel) lines indicate the amplification signals of WT and MT alleles, respectively.

The CRAS-PCR system (Figure 1) is a novel version of AS-PCR in which unidirectional WT- and MT-AS primers (WT-ASF and MT-ASF in the present study) are used in a single tube to eliminate the non-specific amplification commonly occurring in traditional AS-PCR. By nature, the primer pairs in PCR reactions attempt to trigger amplification of input DNA under the pressure of the thermodynamic driving force of thermophilic DNA polymerase. In the AS-PCR system, a defined AS primer has only a single base at its 3′-end that distinguishes the genotype of the input DNA [10], [12], [13], [19], [20]. Accurate annealing between the AS primer and the corresponding complementary input DNA triggers a genotype-specific amplification reaction [19], [20]. However, if only a single genotypic AS primer (e.g., MT genotype) is included in the reaction, under the thermodynamic driving force of DNA polymerase, the single base terminal mismatch between primers and template could easily trigger the non-specific amplification of an input DNA having opposite genotype (e.g., WT genotype) [41]. Moreover, weak-destabilization effects of terminal mismatches could further promote non-specific amplification [42]. Although stringent reaction conditions can be used to dramatically reduce or eliminate non-specific amplification, optimization is time-consuming, and sometimes unsuccessful.

For any given SNP variant, at least one WT- or MT-genotypic allele is present in the AS-PCR system. Therefore, if both primers are present, at least one genotypic AS primer will perfectly match the input DNA with the corresponding genotype and trigger specific amplification. Such amplification would perfectly fulfill the thermodynamic driving force of thermophilic DNA polymerase [41] and thus dramatically reduce or eliminate the weakly destabilizing effects of terminal mismatch [42]. Therefore, for CRAS-PCR, amplification triggered by perfectly matched AS primers always occurs. The availability of specific amplification can dramatically reduce or eliminate the non-specific amplification in traditional AS-PCR systems [10], [12], [19], [20].

Because the amplicon size of different genotypic input DNAs may be the same or indistinguishable by agarose gel electrophoresis, unidirectional competitive AS primers in CRAS-PCR systems are designed as scorpion primers [43], [44]. A unique fluorescent reporter and corresponding quencher is labeled at the 5′- and 3′-end of the stem-loop structure of scorpion primers (Figure 1). Therefore, a defined fluorescent signal can be used to directly identify the genotype of the input DNA (e.g., CY5 fluorescent channel for the WT genotype and 6-FAM fluorescent channel for the MT genotype in the present study; Table 1) [43], [44]. To further distinguish the genotype, additional mismatches can be introduced *penultimate* from the 3′-end for both WT-ASF and MT-ASF primers according to the Web-based Allele-Specific PCR Primer (WASP) principle (Table 1) [42]. Moreover, because distinguishable alleles are present, the fluorescent signal produced by one AS primer could serve as an internal positive control for another. For example, the negative fluorescent signal of the MT-ASF primer might be a true negative if a positive fluorescent signal is provided from the WT-ASF primer (e.g., for WT homozygotes), but might be a false negative if a negative fluorescent signal is provided from WT-ASF primers (e.g., if input DNA was accidently omitted).

### Construction of WT-QC and MT-QC plasmids

**Figure 2:**
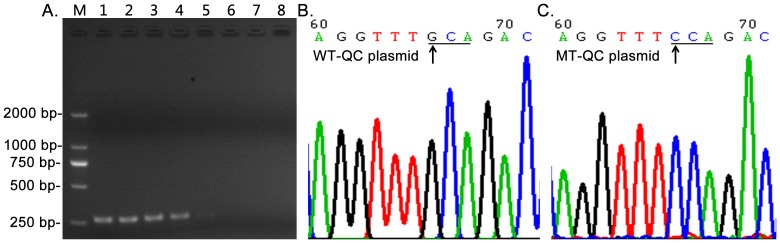
Construction of QC plasmid used in CRAS-PCR. Panel A shows the PCR products of TPMT exon 4 containing the TPMT*2 locus at a 55 to 65°C gradient annealing temperature (lane 1 to 8). Panels B and C show the sequencing chromatogram images of WT- and MT-QC plasmid, in which the arrows indicate the location of TPMT*2, and the underlined bases show the codon containing TPMT*2.

To develop a system for optimization of CRAS-PCR, the PCR products of the TPMT exon 4 locus (Figure 2A) were cloned to prepare the WT-QC plasmid for TPMT*2. This plasmid was further used to prepare a corresponding MT-QC by site-directed mutagenesis. Both plasmids were sequenced to confirm the TPMT*2 genotype (Figure 2B; Figure S1–3). A 1∶1 mixture of above two plasmids was used as MIX-QC plasmids.

### Comparison of traditional AS-PCR and CRAS-PCR by the Taguchi method

According to the principles of the Taguchi method, a modified L_16_ (4^5^) orthogonal array was designed with 16 experiments using four factors with four levels each (Table S1 and S2). Based on the AS primers, the conditions were optimized and characteristics of traditional AS-PCR (Assay Type 1 and 2) and CRAS-PCR (Assay Type 3; Table 2, Table S1 and S2) were evaluated. Moreover, each Assay Type was further subgrouped into three assays based on the genotype of the input DNA (WT-, MT-, or MIX-QC plasmid; Table 2). CY5 and 6-FAM fluorescence signaled the positive amplification of the TPMT*2 WT- or MT-genotype, respectively (Table 1 and 2; Figure 1).

**Figure 3:**
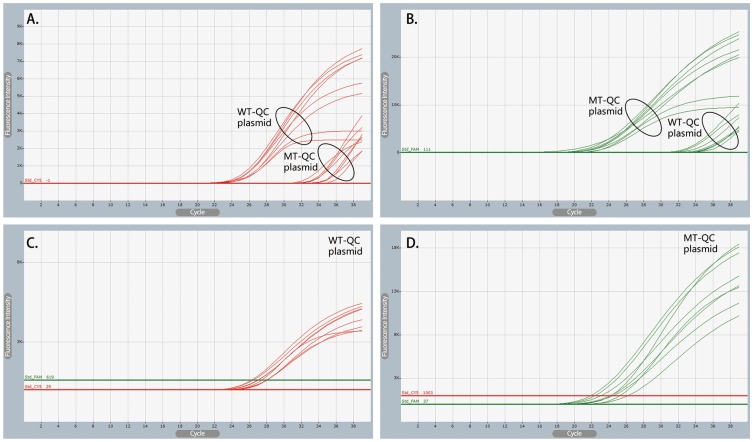
Examples of amplification curves using conditions optimized by the Taguchi Method. Amplification curves of traditional AS-PCR (Panels A and B) and CRAS-PCR (Panels C and D) at 53 °C annealing temperature. Non-specific amplification by traditional AS-PCR was completely eliminated for CRAS-PCR. For Panel A (Assays No. 1-1 and 1-2) and Panel B (Assays No. 2-1 and 2-2), the open circles indicate the amplification curves of corresponding labeled QC plasmids. For Panels C and D (Assay No. 3-1 and 3-2), the corresponding QC plasmids are labeled on the upper right corners.

Assay Types 1 and 2 (Table 2) represent traditional AS-PCR for which only WT- or MT-AS primers were used, and primer extension theoretically only occurs when the 3′-end is perfectly complementary to the allele present in the input sample [19], [20]. However, the results of the Taguchi method shows that non-specific amplification occurred almost uniformly for the MT-QC plasmid in Assay No. 1-2 and for WT-QC plasmid in Assay No. 2-1 (Figure 3A and 3B; Table S4 and S5). This further confirms that the thermodynamic driving force of thermophilic DNA polymerase can promote the non-specific amplification of traditional AS-PCR, despite the additional mismatch in the AS primers [10], [12], [13], [19], [20], [42].

Compared with traditional AS-PCR, all experiments in the CRAS-PCR system (Assay Type 3) showed specific amplification regardless of the genotype of the input DNA (Figure 3C and D, Table S6 and S7). This result further confirms that the thermodynamic driving force of thermophilic DNA polymerase can be perfectly fulfilled in the CRAS-PCR system, thus eliminating the non-specific amplification commonly occurring in traditional AS-PCR.

### Optimization and evaluation of traditional AS-PCR and CRAS-PCR by the Taguchi method

To determine the optimal conditions for traditional AS-PCR and CRAS-PCR, *S*/*N* ratios (*η*) were calculated (Table 3; Table S4 to S7). The mean *η* for each level of each factor was used to determine the optimal level (highest *S*/*N* ratio) and to calculate the *P*_C_ values of each factor (Table 3). The *P*_C_ values reflect the amount of variation of each factor. For traditional PCR (Assay Types 1 and 2), the annealing temperature had the highest *P*_C_ values and significantly contributed to both the amplification efficiency and specificity, for which lower annealing temperatures yielded higher amplification efficiency (Assay No. 1-1 and 2-2), and higher annealing temperatures yielded higher amplification specificity (Assay No. 1-2 and 2-1). Moreover, the AS primer concentration had the highest *P*_C_ values and affected the amplification efficiency and specificity: higher concentrations (Assay No. 1-1 and 2-2) yielded higher efficiency and lower concentrations (Assay No. 1-2 and 2-1) yielded higher specificities. These characteristics are consistent with the common thermodynamic features of traditional AS-PCR. Therefore, the results further confirm that the Taguchi method is useful for understanding the features of molecular analysis methods such as AS-PCR in the current study.

**Table 3:** The average signal-to-noise ratios (*η*) of each level of each factor.

Assay No.	Factor	Level				Optimum	p	*P*_C_ (%)
		1	2	3	4			
1-1	WT-ASF	−28.27	−28.02	−27.88	−27.98	0.6 μM	0.070	28.28
	CO-R	−28.09	−27.92	−28.01	−28.13	0.4 μM	0.370	8.85
	Tm	−27.89	−27.95	−27.95	−28.36	53 °C	0.022*	48.79
	Error							14.08
1-2	WT-ASF	30.72	30.95	31.20	31.38	0.2 μM	0.012*	23.38
	CO-R	31.05	31.02	31.12	31.08	0.6 μM	0.890	0.53
	Tm	30.70	30.91	30.84	31.81	59 °C	0.001*	70.87
								5.22
1-3	WT-ASF	−28.18	−28.07	−28.05	−28.14	0.6 μM	0.040*	10.45
	CO-R	−28.05	−28.11	−28.16	−28.11	0.2 μM	0.103	6.33
	Tm	−27.98	−28.07	−28.04	−28.35	53 °C	0.000*	79.30
	Error							3.92
2-1	WT-ASF	31.21	30.92	31.11	30.98	0.2 μM	0.240	6.26
	CO-R	31.05	31.11	31.01	31.05	0.4 μM	0.888	0.70
	Tm	30.69	30.72	31.09	31.72	59 °C	0.001*	86.24
	Error							6.79
2-2	WT-ASF	−27.28	−26.95	−26.86	−26.78	0.8 μM	0.007*	43.38
	CO-R	−27.04	−27.04	−26.93	−26.87	0.8 μM	0.279	6.34
	Tm	−26.75	−26.90	−26.97	−27.27	53 °C	0.008*	42.50
	Error							7.78
2-3	WT-ASF	−27.20	−27.02	−26.80	−26.90	0.6 μM	0.048*	34.78
	CO-R	−27.01	−27.03	−26.91	−26.97	0.6 μM	0.742	3.03
	Tm	−26.81	−26.93	−26.92	−27.27	53 °C	0.024*	47.92
	Error							14.27
3-1	WT-ASF	−28.58	−28.18	−28.07	−28.16	0.6 μM	0.008*	49.32
	MT-ASF	−28.15	−28.14	−28.35	−28.36	0.4 μM	0.046*	14.19
	CO-R	−28.27	−28.10	−28.18	−28.45	0.4 μM	0.026*	21.86
	Tm	−28.20	−28.21	−28.16	−28.42	57 °C	0.051	13.19
	Error							1.44
3-2	WT-ASF	−27.23	−27.23	−27.38	−27.64	0.2 μM	0.159	17.58
	MT-ASF	−27.82	−27.51	−26.97	−27.19	0.6 μM	0.031*	63.54
	CO-R	−27.28	−27.39	−27.51	−27.30	0.2 μM	0.470	5.33
	Tm	−27.25	−27.26	−27.46	−27.51	53 °C	0.321	8.71
	Error							4.84
3-3	WT-ASF	−11.40	−18.62	−13.25	−14.99	0.2 μM	0.324	12.18
	MT-ASF	−7.81	−10.97	−15.35	−24.13	0.2 μM	0.049*	64.65
	CO-R	−17.22	−11.32	−13.48	−16.23	0.4 μM	0.406	9.24
	Tm	−15.24	−12.60	−12.83	−17.59	55 °C	0.489	7.09
	Error							6.85

*, *p* < 0.05.

The “Smaller-the-Better” equation was used to calculate the *S*/*N* ratios of all assays except for the “Larger-the-Better” equation for Assay No. 1-2 and 2-1, for which *C*_q_ values less than 40 indicate non-specific amplification. The corresponding values at the optimum levels and percent contributions are shown in the “Optimum” and “*P*_C_” column, respectively.

Compared with traditional AS-PCR, in CRAS-PCR the annealing temperature had the lowest *P*_C_ values regardless of the genotype of input DNA, which indicates that the CRAS-PCR maintained specific amplification under relaxed annealing temperature (Table 3). Moreover, for a defined homozygous genotypic input DNA (Assay No. 3-1 and 3-2), the corresponding AS primer had the highest *P*_C_ values and significantly contributed to the amplification efficiency. However, for heterozygous input DNA (Assay No. 3-3), only MT-ASF had significantly higher *P*_C_ values.

Further detailed analysis of Assay Type 3 showed that the optimal CRAS-PCR conditions were dependent on the genotype of the input DNA (Table 3). According to the principle of the Taguchi method and parameters used to calculate *S*/*N* ratios, the optimal levels of each factor reflect the optimal components used for WT and MT homozygotes (Assay No. 3-1 and 3-2, respectively) and heterozygotes (Assay No. 3-3) (Table 2 and 3).

### Evaluation of CRAS-PCR performance following optimization

Both the WT- and MT-alleles of TPMT*2 provide important information for clinical applications. Therefore, an optimal CRAS-PCR method should equivalently amplify each genotypic DNA, either in homozygous or heterozygous state. To further evaluate and confirm the characteristics of the optimized CRAS-PCR system, WT-, MT- and MIX-QC plasmids were used as input DNA for CRAS-PCR performed under the conditions optimized for Assays No. 3-1 to 3-3 (Table 3). The results further confirmed specific amplification for all three assays (Table 4, Table S8). Comparative analysis showed that the optimal level of Assays No. 3-1 and 3-2 had high amplification efficacy for the corresponding genotypic DNA, but not for the opposite genotypic DNA. For Assay No. 3-3, the amplification of heterozygous DNA (MIX-QC plasmid) had a low Δ*C*_q_ value (Δ*C*_q_ = 0.44; SD = 0.24), indicating equivalent amplification of each genotype. Triplicate experiments at optimal levels were performed, and *S*/*N* ratios and mean response were compared with predicted values. All assays had observed *S*/*N* ratios and mean response within the 95%confidence interval (Table 4), indicating that each model was accurate.

**Table 4:** Confirmatory test conditions and corresponding observed *S*/*N* ratios and mean response, predicted *S*/*N* ratios and mean response, and predicted 95% confidence intervals.

	Assay No. 3-1	Assay No. 3-2	Assay No. 3-3
QC Plasmid	WT-QC	MT-QC	MIX-QC
WT-ASF (μM)	0.6	0.2	0.2
MT-ASF (μM)	0.4	0.6	0.2
CO-R (μM)	0.4	0.2	0.4
Tm (°C)	57	53	55
*η* observed	−27.69	−26.56	6.30
*η* predicted	−27.73	−26.62	0.57
*η* prediction Error	−0.06	0.06	5.73
*η* confidence interval	±0.23	±0.61	±13.71
*η* within CI (95%)	Yes^#^	Yes	Yes
Mean response observed*	24.23	21.27	0.44
Mean response predicted	24.29	21.38	0.74
Mean response prediction error	−0.06	−0.11	−0.30
Mean response confidence interval	±0.79	±1.54	±9.32
Mean response within CI (95%)	Yes	Yes	Yes

#. The “Yes” within CI (95%) row indicates that the observed *S*/*N* ratio (*η*) or mean response (*C*_q_ or Δ*C*_q_) was within the corresponding prediction interval.

*. The “mean response” refers to *C*_q_ values in Assay No. 3-1 and 3-2, and Δ*C*_q_ values in Assay No. 3-3.

**Figure 4:**
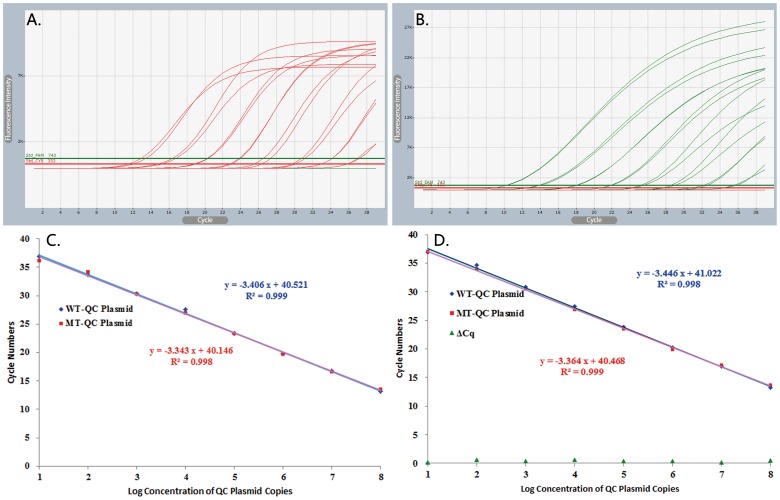
Specificities and calibration plots of CRAS-PCR at optimal levels determined by the Taguchi method. Amplification curves of 10-fold serial dilutions (1×10^8^ to 1×10^1^ copies) of WT- (red lines) and MT-QC (green lines) are shown in Panels A and B, respectively. All assays showed specific amplification of targeted genotypic alleles. Calibration plots of homozygous (i.e., WT- or MT-QC plasmid) and heterozygous (i.e., MIX-QC plasmid) genotype alleles are shown in Panels C and D, respectively. The amplification efficiencies of homozygous WT- and MT-alleles (in Panel C) were 96.59% (slope: −3.406; R^2^ = 0.999) and 99.11% (slope: −3.343; R^2^ = 0.998), respectively. The amplification efficiencies of WT- and MT-alleles in heterozygous samples (in Panel D) were 95.07% (slope: −3.446; R^2^ = 0.998) and 98.27% (slope: −3.364; R^2^ = 0.999), respectively. The average Δ*C*_q_ values of WT- and MT-alleles in heterozygous samples (Panel D) were 0.42 (SD: 0.19).

Based on above results, the reaction conditions of Assay No. 3-3 (0.2 μM each of WT- and MT-ASF primers, 0.4 μM of CO-R primers, and annealing temperature at 55 °C) were confirmed as the optimal parameters to analyze the TPMT*2 genotype. Ten-fold serially diluted WT-, MT- and MIX-QC plasmid (i.e., 1×10^8^ to 1×10^1^ copies of each type of plasmid) was used to further evaluate features of the above optimized CRAS-PCR system. Using a conversion factor of 6.6 pg per diploid human cell [39], [40], this is equal to 330 μg to 33 pg of human genomic DNA for WT- and MT-QC plasmid, and μg to 66 pg for MIX-QC plasmid in μl of reaction mixture, which replicates the human genomic DNA concentration used in real-time PCR. For homozygous genotypic DNA (i.e., WT- or MT-QC plasmid) at 10-fold serial dilution concentrations (1×10^8^ to 1×10^1^ copies), specific amplification consistently occurred (Figure 4A, B), which further confirms the specificity and selectivity of the optimized CRAS-PCR system. For the amplification efficiencies of homozygous (i.e., WT- or MT-QC plasmid) or heterozygous (i.e., MIX-QC plasmid) genotypic DNA, the WT- and MT-alleles had equivalent amplification efficiencies and *C*_q_ values regardless of the genotype of input DNA (Figure 4C, D). Furthermore, the CRAS-PCR system specifically determined the genotype of input homozygous DNA at a limitation of 10 copies, demonstrating a high level of sensitivity. Consistent with the data from Table 4, the average Δ*C*_q_ value (0.42) for the serially diluted MIX-QC plasmid fell within the predicted 95% CI, further verifying the accuracy of the model.

**Figure 5:**
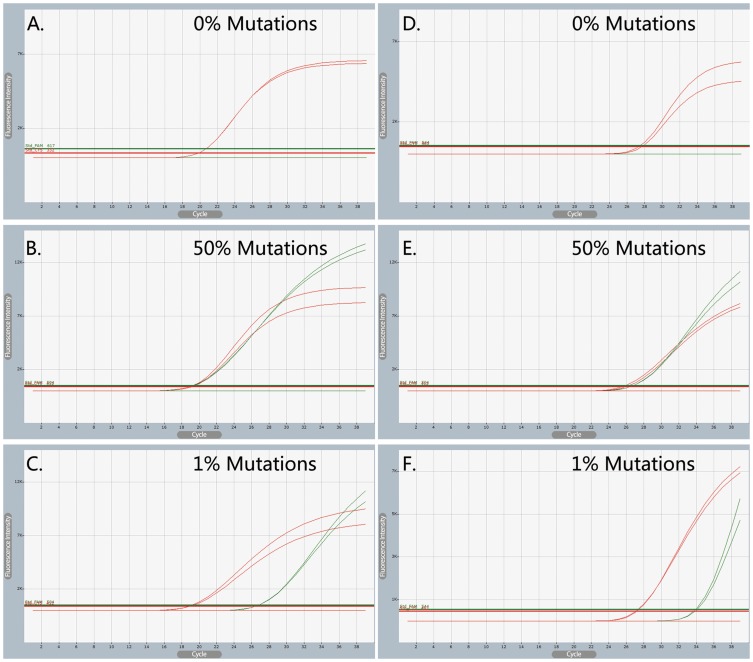
Genotyping sensitivities of CRAS-PCR at optimal levels determined by the Taguchi method. The concentration of WT-QC plasmid (red lines) was 1×10^6^ copies in Panel A to C and 1×10^4^ copies in Panel D to F. The mutation percentage of MT-QC plasmid (green lines) in each assay is indicated as labeled, in which 0% mutations indicates homozygous input DNA (i.e., WT-QC plasmid); 50% mutations indicates 1×10^6^ copies in Panel B and 1×10^4^ copies in Panel E; and 1% mutations indicates 1×10^4^ copies in Panel C and 1×10^2^ copies in Panel F. The results of other combinations of WT- and MT-QC plasmid showed equivalent results, i.e., 1% sensitivity of mutation analysis (data not shown).

To further evaluate the ability to detect low-frequency or rare mutant alleles, various concentrations of MT-QC plasmid (1×10^6^ to 1×10^1^ copies) were mixed with serially diluted WT-QC plasmid (1×10^6^ to 1×10^1^ copies). The results show that the current CRAS-PCR system has ability to distinguish allelic differences at sensitivities of 1% regardless of the concentration of input heterozygous DNA (Figure 5). For example, when the input WT-QC plasmid is at 1×10^6^ copies, the current CRAS-PCR system has the ability to detect 1×10^4^ copies of MT-QC plasmid although the current CRAS-PCR system has the ability to detect as low as 10 copies of mutant allele without the interference of high-abundant WT-alleles (Figure 4). The ability to distinguish alleles (i.e., 1%) is similar to traditional AS-PCR) [45], [46]. This result indicates that the higher abundance of certain genotypic DNA would suppress or eliminate low-frequency or rare opposite genotypic DNA.

### TPMT*2 frequencies in Chinese analyzed by CRAS-PCR

**Figure 6:**
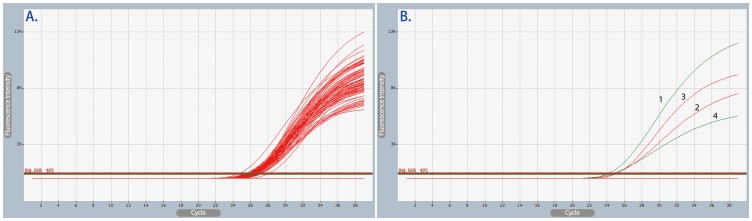
Examples of CRAS-PCR amplification curves under conditions optimized by the Taguchi Method. Examples of amplification curves of clinical samples (Panel A) and corresponding QC plasmids (Panel B) in which the CY5 (red lines) and 6-FAM (green lines) fluorescent channel indicate WT- and MT-genotype signals, respectively. In Panel B, the amplification curves for WT- (labeled with 1), MT- (labeled with 2) and MIX- (labeled with 3 and 4) QC plasmid are shown as a control.

To verify the specificity for clinical application of the optimized CRAS-PCR system, the optimal levels of the four factors in Assay Types No. 3-3 were further used to analyze the TPMT*2 genotype of 240 clinical blood samples from ethnic Chinese. This population is known to be almost exclusively WT [11], [47], and therefore, a predominant lack of positivity for the MT allele would rule out the possibility of non-specific amplification by the MT-ASF primer. Each sample was analyzed in duplicate, and WT-, MT- and MIX-QC plasmid were tested in parallel to monitor the amplification quality. The results showed that, as expected, all genotypes were WT, with no MT or heterozygous genotype samples (Figure 6). These results were further demonstrated by DNA sequencing of exon 4 fragments containing TPMT*2. Thus, this assay demonstrates the effective application of CRAS-PCR optimized by the Taguchi method for genotype determination of clinical DNA samples.

## Discussion

We have provided a comprehensive analysis of the differences between AS-PCR and CRAS-PCR and have also described efficient methodology for evaluation and optimization of each approach. In traditional AS-PCR, AS primers or AS probes are used to analyze the genotype of input DNA [10], [11], [12], [13], [14], [15], [19], [20]. At least one of the primer pairs targeting variant sequences is chosen from a polymorphic area with mutations located at or near its 3′-end. However, successful genotype determination is dependent on stringent conditions (annealing temperature, and primer and Mg^2+^ concentrations) [10], [11], [19], [20], [42], which could affect the sensitivity; additionally, the specificity is often hampered by cross-annealing, even when stringent reaction conditions are used [17], [18], [42]. Moreover, because the amplicon fragment size is often the same, two separate reactions are used to analyze the WT and MT genotype of input DNA [19], [20], [43], [44]. By nature, if primer pairs and corresponding templates are present, the PCR system tries to produce amplicons due to the thermodynamic driving force of thermophilic DNA polymerase [41], often leading to non-specific amplification in AS-PCR [10], [11], [19], [20], [42]. However, because CRAS-PCR uses both primers in a single reaction, for any defined input DNA a specific amplification reaction will occur, satisfying the thermodynamic driving force and reducing nonspecific amplification (Figure 1). The design parameters for CRAS-PCR outlined in this study demonstrate the advantage of the CRAS-PCR system for the avoidance of nonspecific amplification (Figure 3).

Regardless of the methodology for PCR, the amplification of input DNA by thermophilic DNA polymerase depends on reaction components (e.g., primer or Mg^2+^ concentrations) and thermal cycling parameters (e.g., annealing temperature and duration); therefore, reproducibility and accuracy is contingent on adequate optimization [41]. Optimization demands an investigation of the interaction between multiple variables and involves unusually large experiments [31], [41]. Traditionally, complete optimization can be achieved by testing each variable component independently by the factorial method, which is costly and time-consuming [31]. However, in the current study, the Taguchi method, a Design of Experiment (DOE) [21], [22], [23] was used to optimize conditions and evaluate characteristics of AS-PCR and CRAS-PCR. The Taguchi method was selected because it provides a systematic approach towards designing experiments [21], [22], [23], and consequently has been the most widely used technique in automotive and electronics industrial design for the past two decades [21], [22], [23], [28], [32], [33], [34], [35]. One of the important steps in Taguchi's technique is the selection of orthogonal arrays, which helps to determine the optimum level for each parameter and establishes the relative importance of individual parameters using a small set of all possibilities in a minimal number of experiments [21], [22], [23]. In the present study, according to the principle of the Taguchi method, a modified L_16_(4^5^) orthogonal array was selected to optimize conditions and evaluate characteristics of both traditional AS-PCR and CRAS-PCR. Compared with factorial methods requiring 64 (4^3^) experiments for traditional AS-PCR (Assay Types 1 and 2) and 256 (4^4^) experiments for CRAS-PCR (Assay Type 3), only 16 experiments were need for each above assay, which dramatically increased the efficiency of optimization and evaluation.

The effectiveness of the Taguchi method was demonstrated using a variety of statistical methods. In all assays of the Taguchi method, the *P*_C_ values of errors were less than 15% (Table 3), which indicated the acceptable reliability of orthogonal array selection, and significant factors were analyzed in each assay [21], [22], [23]. In assays of traditional AS-PCR (Assay Type 1 and 2), both “smaller-the-better” and “larger-the-better” strategies were selected in which the *C*_q_ values served as response variables. The annealing temperature had the highest *P*_C_ values and its *p* values were less than 0.05. This result indicates that the annealing temperature had significant effect on both amplification efficiency and specificity for traditional AS-PCR. We aimed to determine the contribution of higher and lower concentrations of AS primers for efficiency (Assay No. 1-1 and 2-2 in Table 3) and specificity (Assay No. 1-2 and 2-1 in Table 3). The results indicated that both higher annealing temperature and lower AS primer concentrations contribute to the specificity of AS-PCR. Among the two factors, the annealing temperature was the most important parameter because it had significant higher *P*_C_ values (Table 3).

For CRAS-PCR, the “smaller-the-better” strategy was used in which the *C*_q_s or Δ*C*_q_s were used as response variables based on the genotype of the input DNA. Compared with the results of traditional AS-PCR, the annealing temperature almost always had lower *P*_C_ values, and did not significantly affect the efficiency or specificity of the CRAS-PCR system. Moreover, for input DNA having homozygous genotype (Assay No. 3-1 and 3-1 in Table 3), the concentration of the corresponding AS primers had the highest *P*_C_ values. However, using the Δ*C*_q_ as a response variable for input DNA having heterozygous genotype (Assay No. 3-3 in Table 3), only MT-ASF primers had higher *P*_C_ values, which indicates that the concentration of MT-ASF primers had more effect on the uniform amplification of both WT- and MT-genotypic input DNA (Table S6). Notably, compared with traditional PCR (Assay Type 1 and 2) in which non-specific amplification is present in almost all experiments, there was no non-specific amplification in any experiments for the CRAS-PCR system (S4 to S6). Therefore, the non-specific amplification of the traditional AS-PCR system can be eliminated or dramatically decreased if both genotypic primers are used in a single tube, such as in the CRAS-PCR system developed in the current study.

Among various single nucleotide mismatches, GA, CT, TT and CC mismatches almost always have strong destabilization strength, AA and GG mismatches have medium destabilization strength, and CA and GT mismatches have weak destabilization strength [42], [48]. In many cases, a single nucleotide mismatch at the 3′-end of the AS primer is not enough to specifically distinguish mutant alleles from wild-type ones. As a general rule, an additional mismatch can be introduced into the 3′-terminal position to enhance the destabilization strength of the AS primers to provide greater ability to distinguish alleles in AS-PCR systems. For example, if the 3′-terminal mismatch is a weak one (e.g., GT mismatch), a strong *penultimate* mismatch could be engineered, and *vice versa* [42], [48]. In our preliminary experiments, two strategies were used to design WT- and MT-ASF primers: one was fully complementary to the corresponding genotype alleles; another manually introduced a mismatched nucleotide at the *penultimate* position according to the WASP principle [42]. Compared with fully complementary AS primers, the currently designed AS primers (i.e., WT- and MT-ASF) consistently showed higher specificities in traditional AS-PCR systems (data not shown). The results of the Taguchi method indicate that the CRAS-PCR system using AS primers designed according to the WASP principle almost had annealing temperature-independent specificity and selectivity, which suggests that the design strategies of AS primers are crucial to such a feature of the CRAS-PCR system. However, the current study just evaluates the allele-distinguishing abilities of the CRAS-PCR system to specifically identify polymorphisms having strong (i.e., CC mismatch) to medium (i.e., GG mismatch) destabilization effects. Another thorough study should be further performed to analyze the ability to identify AS primers having only weak destabilization effects, such as CA and GT mismatches, in the CRAS-PCR system.

Based on the results of the Taguchi method and the final objective of TPMT*2 genotyping, optimal levels of four factors were used to evaluate the genotype of 240 clinical samples. The frequency of TPMT*2 genotype was essentially similar to previous publications, which indicated that TPMT*3C was found in 2.3% Chinese, but TPMT*2, *3A and *3B were not detected [11], [47]. Based on the frequency of TPMT*2, *3A, *3B and *3C in ethnic Chinese, a CRAS-PCR system targeting TPMT*3C is being developed in our laboratory according to the principles described in the current study.

In summary, a novel type of AS-PCR, CRAS-PCR, was developed in the present study. The Taguchi method was used to optimize conditions and evaluate characteristics of both traditional AS-PCR and CRAS-PCR. Compared with factorial methods, the Taguchi method provided a powerful statistic approach to determine optimal factor levels and understand characteristics. Compared with traditional AS-PCR, CRAS-PCR completely eliminated non-specific amplification at more relaxed conditions, which suggests that CRAS-PCR could serve as a powerful and robust routine genotyping tool for SNPs analysis, including TPMT*2, in a single tube.

## Supporting Information

Figure S1 **Sequencing Chromatograph of WT-QC Plasmid.**
(PDF)

Figure S2 **Sequencing chromatograph of MT-QC plasmid.**
(PDF)

Figure S3 **Sequencing alignment between the reference genomic sequence and QC plasmids.**
(PDF)

Table S1 **Four factors and four levels for corresponding assays.**
(PDF)

Table S2 **The modified L_16_(4^5^) orthogonal array used to optimize traditional AS-PCR (Assay Types 1 and 2).**
(PDF)

Table S3 **The modified L_16_(4^5^) orthogonal array used to optimize CRAS-PCR (Assay Type 3).**
(PDF)

Table S4 **Quantification cycles of duplicate runs (** ***C*** **_q_1 and** ***C*** **_q_2) for all experiments and their corresponding** ***S*** **/** ***N*** **ratio (** ***η*** **) in Assay Type 1.**
(PDF)

Table S5 **Quantification cycles of duplicate runs (** ***C*** **_q_1 and** ***C*** **_q_2) for all experiments and their corresponding** ***S*** **/** ***N*** **ratio (** ***η*** **) in Assay Type 2.**
(PDF)

Table S6 **Quantification cycles of duplicate runs (** ***C*** **_q_1 and** ***C*** **_q_2) for all experiments and their corresponding** ***S/N*** **ratio (** ***η*** **) in Assay No. 3-1 and 3-2.**
(PDF)

Table S7 **Quantification cycles of quadruplet runs (** ***C*** **_q_1 to** ***C*** **_q_4) for all experiments and their corresponding** ***S/N*** **ratio (** ***η*** **) of each sample Δ** ***C*** **_q_ in Assay No. 3-3.**
(PDF)

Table S8 **Quantification cycles of triplicate runs (** ***C*** **_q_1 to** ***C*** **_q_3) for confirmatory experiments and their corresponding** ***S/N*** **ratio (** ***η*** **) in Assay Type 3 (CRAS-PCR).**
(PDF)
