# Supplementary material for: Enhanced Specificity of TPMT*2 Genotyping Using Unidirectional Wild-Type and Mutant Allele-Specific Scorpion Primers in a Single Tube
Source: PLoS One. 2014 Apr 4;9(4):e91824. doi: 10.1371/journal.pone.0091824 (PMC3976262; doi:10.1371/journal.pone.0091824)
Supplement: Figure S1 — Sequencing Chromatograph of WT-QC Plasmid. (PDF) [file pone.0091824.s001.pdf]

Figure S1. Sequencing Chromatograph of WT-QC Plasmid

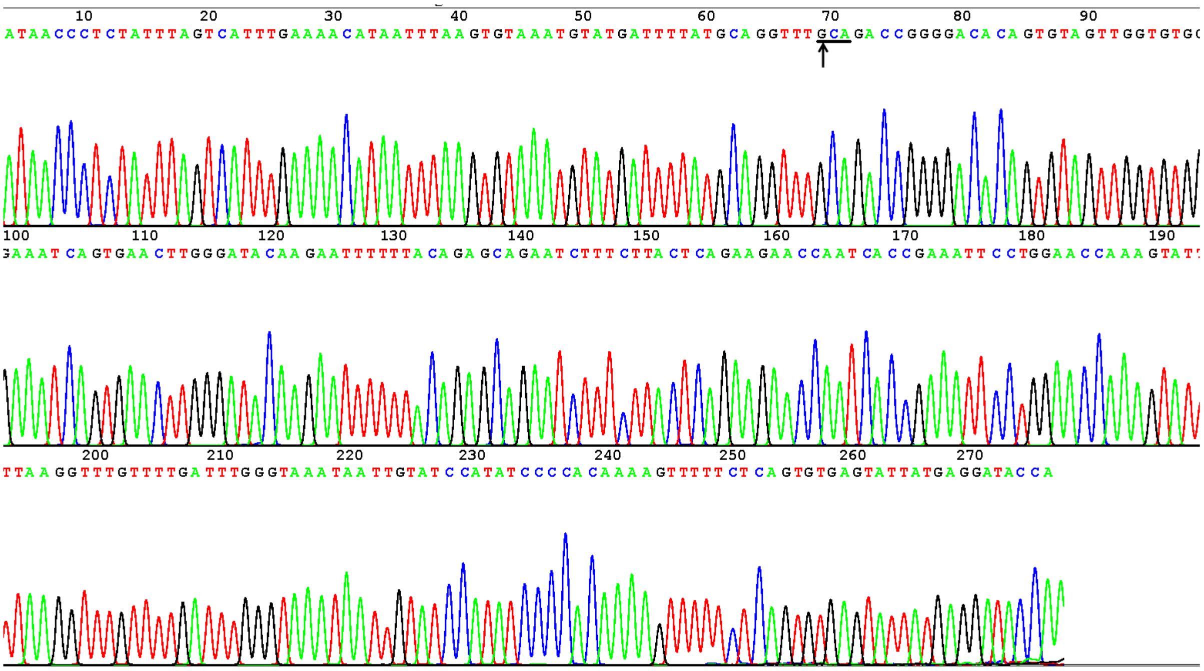

The arrow indicates the location of TPMT*2, and the underlined bases show the codon containing TPMT*2.
