# Supplementary material for: Enhanced Specificity of TPMT*2 Genotyping Using Unidirectional Wild-Type and Mutant Allele-Specific Scorpion Primers in a Single Tube
Source: PLoS One. 2014 Apr 4;9(4):e91824. doi: 10.1371/journal.pone.0091824 (PMC3976262; doi:10.1371/journal.pone.0091824)
Supplement: Figure S3 — Sequencing alignment between the reference genomic sequence and QC plasmids. (PDF) [file pone.0091824.s003.pdf]

Figure S3. Sequencing alignment between the reference genomic sequence and QC plasmids

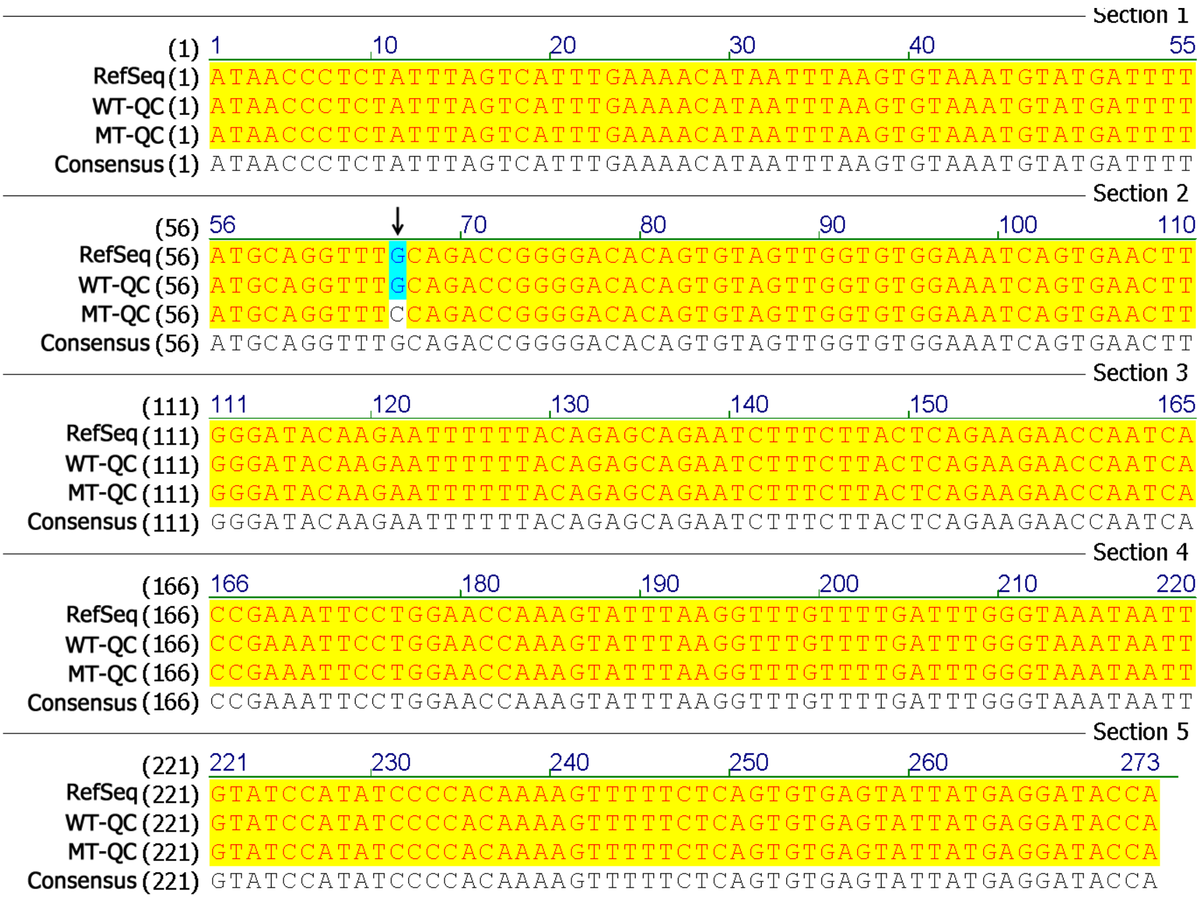

The RefSeq refer to the reference genomic sequence (NCBI GenBank ID: NG_012137.1) amplified by HQ-489 and HQ-490. The black arrow indicates the position of TPMT*2.
