# Supplementary material for: Enhanced Specificity of TPMT*2 Genotyping Using Unidirectional Wild-Type and Mutant Allele-Specific Scorpion Primers in a Single Tube
Source: PLoS One. 2014 Apr 4;9(4):e91824. doi: 10.1371/journal.pone.0091824 (PMC3976262; doi:10.1371/journal.pone.0091824)
Supplement: Table S1 — Four factors and four levels for corresponding assays. (PDF) [file pone.0091824.s004.pdf]

Table S1. Four factors and four levels for corresponding assays

Factor	Level 1	Level 2	Level 3	Level 4
Wild-type alleles-specific forward primer (WT-ASF; μM)	0.2	0.4	0.6	0.8
Mutant-type alleles-specific forward primer (MT-ASF; μM)	0.2	0.4	0.6	0.8
Common reverse primer (CO-R; μM)	0.2	0.4	0.6	0.8
Annealing temperature (Tm; °C)	53	55	57	59
