# Supplementary material for: Enhanced Specificity of TPMT*2 Genotyping Using Unidirectional Wild-Type and Mutant Allele-Specific Scorpion Primers in a Single Tube
Source: PLoS One. 2014 Apr 4;9(4):e91824. doi: 10.1371/journal.pone.0091824 (PMC3976262; doi:10.1371/journal.pone.0091824)
Supplement: Table S2 — The modified L16(45) orthogonal array used to optimize traditional AS-PCR (Assay Types 1 and 2). (PDF) [file pone.0091824.s005.pdf]

Table S2. The modified $L_{16}(4^5)$ orthogonal array used to optimize traditional AS-PCR (Assay Types 1 and 2)

	Level		
Experiment	WT-ASF (or MT-ASF)	CO-R	Tm
1	1	1	1
2	1	2	2
3	1	3	3
4	1	4	4
5	2	1	2
6	2	2	1
7	2	3	4
8	2	4	3
9	3	1	3
10	3	2	4
11	3	3	1
12	3	4	2
13	4	1	4
14	4	2	3
15	4	3	2
16	4	4	1

The numbers 1, 2, 3 and 4 indicate the levels tested for each experiment corresponding to Supp. Table 1.
