# Supplementary material for: Enhanced Specificity of TPMT*2 Genotyping Using Unidirectional Wild-Type and Mutant Allele-Specific Scorpion Primers in a Single Tube
Source: PLoS One. 2014 Apr 4;9(4):e91824. doi: 10.1371/journal.pone.0091824 (PMC3976262; doi:10.1371/journal.pone.0091824)
Supplement: Table S3 — The modified L16(45) orthogonal array used to optimize CRAS-PCR (Assay Type 3). (PDF) [file pone.0091824.s006.pdf]

Table S3. The modified $L_{16}(4^5)$ orthogonal array used to optimize CRAS-PCR (Assay Type 3)

	Level			
Experiment	WT-ASF	MT-ASF	CO-R	Tm
1	1	1	1	1
2	1	2	2	2
3	1	3	3	3
4	1	4	4	4
5	2	1	2	3
6	2	2	1	4
7	2	3	4	1
8	2	4	3	2
9	3	1	3	4
10	3	2	4	3
11	3	3	1	2
12	3	4	2	1
13	4	1	4	2
14	4	2	3	1
15	4	3	2	4
16	4	4	1	3

The number 1, 2, 3 and 4 indicated the levels tested for each experiment in Supp. Table 1.
