# Supplementary material for: Enhanced Specificity of TPMT*2 Genotyping Using Unidirectional Wild-Type and Mutant Allele-Specific Scorpion Primers in a Single Tube
Source: PLoS One. 2014 Apr 4;9(4):e91824. doi: 10.1371/journal.pone.0091824 (PMC3976262; doi:10.1371/journal.pone.0091824)
Supplement: Table S4 — Quantification cycles of duplicate runs ( C q1 and C q2) for all experiments and their corresponding S / N ratio ( η ) in Assay Type 1. (PDF) [file pone.0091824.s007.pdf]

Table S4. Quantification cycles of duplicate runs (C_q1 and C_q2) for all experiments and their corresponding *S*/*N* ratio (η) in Assay Type 1

	CY5 Channel signal (TPMT*2 wild-type allele signal)								
	WT-QC Plasmid (Assay No. 1-1)			MT-QC Plasmid (Assay No. 1-2)			MIX-QC Plasmid (Assay No. 1-13)		
Exp.	C_q1	C_q2	η	C_q1	C_q2	η	C_q1	C_q2	η
1	25.02	25.32	-28.02	32.55	33.64	30.39	25.06	24.72	-27.92
2	25.28	25.27	-28.05	33.01	33.28	30.41	25.92	25.37	-28.18
3	26.41	25.31	-28.25	33.86	33.58	30.56	25.75	25.64	-28.20
4	27.37	27.38	-28.75	ND	35.73	31.52	26.64	26.11	-28.42
5	25.20	24.86	-27.97	34.23	34.33	30.70	25.15	24.92	-27.97
6	25.17	24.64	-27.93	33.42	35.03	30.68	25.01	25.02	-27.96
7	25.72	25.22	-28.12	36.63	ND	31.64	26.16	26.24	-28.37
8	25.43	25.16	-28.06	34.42	34.87	30.79	24.89	25.17	-27.97
9	24.59	24.62	-27.82	35.02	36.48	31.06	24.84	25.20	-27.97
10	25.29	25.16	-28.04	ND	ND	32.04	25.98	25.93	-28.28
11	24.46	24.47	-27.77	33.70	35.16	30.73	25.04	24.97	-27.96
12	24.61	24.97	-27.89	35.70	35.05	30.97	25.04	25.19	-28.00
13	25.44	27.93	-28.53	ND	ND	32.04	26.13	26.11	-28.34
14	24.28	24.12	-27.68	35.95	34.55	30.94	25.18	25.21	-28.03
15	24.40	25.13	-27.88	35.91	ND	31.55	25.43	25.59	-28.13
16	24.14	25.18	-27.84	36.07	35.02	31.01	25.17	25.42	-28.06
