# Supplementary material for: Enhanced Specificity of TPMT*2 Genotyping Using Unidirectional Wild-Type and Mutant Allele-Specific Scorpion Primers in a Single Tube
Source: PLoS One. 2014 Apr 4;9(4):e91824. doi: 10.1371/journal.pone.0091824 (PMC3976262; doi:10.1371/journal.pone.0091824)
Supplement: Table S5 — Quantification cycles of duplicate runs ( C q1 and C q2) for all experiments and their corresponding S / N ratio ( η ) in Assay Type 2. (PDF) [file pone.0091824.s008.pdf]

Table S5. Quantification cycles of duplicate runs (*C_q*1 and *C_q*2) for all experiments and their corresponding *S*/*N* ratio (*η*) in Assay Type 2

	CY5 Channel signal (i.e., TPMT*2 wild-type allele signal)								
	WT-QC Plasmid (Assay No. 2-1)			MT-QC Plasmid (Assay No. 2-2)			MIX-QC Plasmid (Assay No. 2-3)		
Exp.	C_q1	C_q2	η	C_q1	C_q2	η	C_q1	C_q2	η
1	34.43	34.88	30.79	23.08	23.05	-27.26	22.70	22.47	-27.08
2	34.97	35.31	30.92	23.20	22.69	-27.21	22.74	22.68	-27.12
3	36.41	35.32	31.09	23.15	23.16	-27.29	23.09	22.59	-27.17
4	ND	ND	32.04	23.59	23.11	-27.37	23.45	23.53	-27.42
5	33.14	34.49	30.58	22.05	21.98	-26.85	22.04	22.21	-26.90
6	32.97	35.64	30.69	21.95	21.98	-26.83	22.57	22.44	-27.05
7	36.98	37.35	31.40	22.76	23.07	-27.20	23.11	23.00	-27.26
8	36.08	35.16	31.03	22.11	22.27	-26.92	22.25	22.00	-26.90
9	35.76	35.95	31.09	22.11	21.72	-26.82	22.00	22.02	-26.85
10	36.99	ND	31.69	23.20	22.99	-27.27	22.96	22.76	-27.18
11	35.04	35.12	30.90	20.95	21.44	-26.53	21.61	19.92	-26.35
12	33.80	35.30	30.76	21.87	22.01	-26.82	22.18	21.73	-26.83
13	ND	37.49	31.75	23.07	22.87	-27.22	22.70	23.22	-27.22
14	37.15	35.21	31.16	22.00	22.01	-26.85	21.58	21.97	-26.76
15	33.32	34.77	30.64	21.72	21.49	-26.69	22.07	22.07	-26.88
16	33.60	32.43	30.37	19.46	22.12	-26.37	21.47	22.04	-26.75
