# Supplementary material for: Enhanced Specificity of TPMT*2 Genotyping Using Unidirectional Wild-Type and Mutant Allele-Specific Scorpion Primers in a Single Tube
Source: PLoS One. 2014 Apr 4;9(4):e91824. doi: 10.1371/journal.pone.0091824 (PMC3976262; doi:10.1371/journal.pone.0091824)
Supplement: Table S6 — Quantification cycles of duplicate runs ( C q1 and C q2) for all experiments and their corresponding S/N ratio ( η ) in Assay No. 3-1 and 3-2. (PDF) [file pone.0091824.s009.pdf]

Table S6. Quantification cycles of duplicate runs (C_q1 and C_q2) for all experiments and their corresponding *S*/*N* ratio (η) in Assay No. 3-1 and 3-2

	WT-QC Plasmid (Assay No. 3-1)						MT-QC Plasmid (Assay No. 3-2)					
	CY5 Channel (WT signal)			6-FAM Channel (MT signal)			CY5 Channel (WT signal)			6-FAM Channel (MT signal)		
Exp.	C_q1	C_q2	η	C_q1	C_q2	η	C_q1	C_q2	η	C_q1	C_q2	η
1	26.08	26.61	-28.41	ND	ND	NA	ND	ND	NA	24.52	23.45	-27.60
2	25.94	25.96	-28.28	ND	ND	NA	ND	ND	NA	22.71	23.04	-27.19
3	26.86	26.37	-28.50	ND	ND	NA	ND	ND	NA	22.36	22.79	-27.07
4	28.95	28.22	-29.12	ND	ND	NA	ND	ND	NA	22.51	22.55	-27.06
5	24.71	24.92	-27.89	ND	ND	NA	ND	ND	NA	24.29	24.40	-27.73
6	25.72	25.99	-28.25	ND	ND	NA	ND	ND	NA	23.11	23.92	-27.43
7	26.36	26.42	-28.43	ND	ND	NA	ND	ND	NA	21.03	21.48	-26.55
8	25.11	25.99	-28.15	ND	ND	NA	ND	ND	NA	22.80	23.12	-27.22
9	25.05	25.54	-28.06	ND	ND	NA	ND	ND	NA	25.32	25.03	-28.02
10	25.14	25.34	-28.04	ND	ND	NA	ND	ND	NA	24.19	24.19	-27.67
11	25.95	25.47	-28.20	ND	ND	NA	ND	ND	NA	22.04	21.26	-26.71
12	24.79	25.32	-27.98	ND	ND	NA	ND	ND	NA	22.16	23.18	-27.11
13	25.85	25.62	-28.21	ND	ND	NA	ND	ND	NA	22.49	27.03	-27.91
14	24.74	25.47	-28.00	ND	ND	NA	ND	ND	NA	23.49	25.21	-27.74
15	25.96	25.74	-28.25	ND	ND	NA	ND	ND	NA	23.86	23.82	-27.55
16	25.05	26.33	-28.20	ND	ND	NA	ND	ND	NA	23.61	23.14	-27.38
