# Supplementary material for: Enhanced Specificity of TPMT*2 Genotyping Using Unidirectional Wild-Type and Mutant Allele-Specific Scorpion Primers in a Single Tube
Source: PLoS One. 2014 Apr 4;9(4):e91824. doi: 10.1371/journal.pone.0091824 (PMC3976262; doi:10.1371/journal.pone.0091824)
Supplement: Table S7 — Quantification cycles of quadruplet runs ( C q1 to C q4) for all experiments and their corresponding S/N ratio ( η ) of each sample Δ C q in Assay No. 3-3. (PDF) [file pone.0091824.s010.pdf]

Table S7. Quantification cycles of quadruplet runs (C_q1 to C_q4) for all experiments and their corresponding *S*/*N* ratio (*η*) of each sample ΔC_q in Assay No. 3-3

	MIX-QC Plasmid (Assay No. 3-3)												
	CY5 Channel (WT signal)				6-FAM Channel (MT signal)				Δ*C*q Values of each sample				
Exp.	C_q1	C_q2	C_q3	C_q4	C_q1	C_q2	C_q3	C_q4	ΔC_q1	ΔC_q2	ΔC_q3	ΔC_q4	*η*
1	26.11	25.83	25.99	26.12	28.48	27.33	28.53	28.64	2.37	1.50	2.54	2.52	-7.13
2	26.13	27.34	26.59	27.80	27.11	26.31	25.19	26.51	0.98	1.03	1.40	1.29	-1.50
3	28.34	28.01	29.05	30.21	24.55	24.09	24.59	25.05	3.79	3.92	4.46	5.16	-12.80
4	ND	ND	ND	ND	23.58	24.22	23.59	24.05	16.42	15.78	16.41	15.95	-24.16
5	25.24	25.29	26.18	25.65	23.19	23.67	24.02	24.12	2.05	1.62	2.16	1.53	-5.39
6	ND	ND	ND	ND	23.52	23.54	23.97	24.51	16.48	16.46	16.03	15.49	-24.15
7	35.32	35.56	35.48	35.89	24.84	24.39	24.38	25.54	10.48	11.17	11.10	10.35	-20.65
8	ND	ND	ND	ND	23.58	23.57	23.38	23.94	16.42	16.43	16.62	16.06	-24.29
9	27.75	27.19	28.52	28.45	25.48	25.78	26.07	25.53	2.27	1.41	2.45	2.92	-7.34
10	26.68	27.45	26.11	26.55	23.96	24.78	23.40	23.70	2.72	2.67	2.71	2.85	-8.75
11	28.04	29.23	28.65	29.35	24.41	24.52	23.67	24.44	3.63	4.71	4.98	4.91	-13.24
12	ND	ND	ND	ND	24.97	24.46	24.24	25.19	15.03	15.54	15.76	14.81	-23.69
13	28.45	28.20	28.92	28.47	24.64	24.64	24.53	25.59	3.81	3.56	4.39	2.88	-11.36
14	25.60	25.89	25.45	27.12	22.67	22.60	23.09	23.85	2.93	3.29	2.36	3.27	-9.50
15	28.99	28.32	27.98	28.23	23.01	22.68	22.83	23.29	5.98	5.64	5.15	4.94	-14.72
16	ND	ND	ND	ND	23.22	23.07	23.88	23.71	16.78	16.93	16.12	16.29	-24.37
