# Supplementary material for: Enhanced Specificity of TPMT*2 Genotyping Using Unidirectional Wild-Type and Mutant Allele-Specific Scorpion Primers in a Single Tube
Source: PLoS One. 2014 Apr 4;9(4):e91824. doi: 10.1371/journal.pone.0091824 (PMC3976262; doi:10.1371/journal.pone.0091824)
Supplement: Table S8 — Quantification cycles of triplicate runs ( C q1 to C q3) for confirmatory experiments and their corresponding S/N ratio ( η ) in Assay Type 3 (CRAS-PCR). (PDF) [file pone.0091824.s011.pdf]

Table S8. Quantification cycles of triplicate runs (C_q1 to C_q3) for confirmatory experiments and their corresponding *S*/*N* ratio (*η*) in Assay Type 3 (CRAS-PCR)

Assay No.	QC Plasmid	CY5 Channel (WT Signal)				6-FAM Channel (MT Signal)			
		C_q1	C_q2	C_q3	η	C_q1	C_q2	C_q3	η
3-1	WT-QC	23.99	24.31	24.39	-27.69	ND	ND	ND	NA
	MT-QC	ND	ND	ND	NA	22.87	23.63	23.36	-27.34
	MIX-QC	25.71	25.56	25.28	-28.14	23.62	23.87	23.34	-27.46
3-2	WT-QC	29.30	28.78	28.86	-29.24	ND	ND	ND	NA
	MT-QC	ND	ND	ND	NA	21.19	21.92	20.71	-26.56
	MIX-QC	ND	ND	ND	NA	24.52	25.05	25.6	-27.98
3-3	WT-QC	24.69	24.45	24.36	-27.78	ND	ND	ND	NA
	MT-QC	ND	ND	ND	NA	24.63	24.17	24.24	-27.73
	MIX-QC	26.25	26.51	26.55	-28.44	25.64	25.96	26.38	-28.30
